# The effect of short-course antibiotics on the resistance profile of colonizing gut bacteria in the ICU: a prospective cohort study

**DOI:** 10.1186/s13054-020-03061-8

**Published:** 2020-07-09

**Authors:** Christian Munck, Ravi U. Sheth, Edward Cuaresma, Jessica Weidler, Stephania L. Stump, Philip Zachariah, David H. Chong, Anne-Catrin Uhlemann, Julian A. Abrams, Harris H. Wang, Daniel E. Freedberg

**Affiliations:** 1 Department of Systems Biology, Columbia University Irving Medical Center, 3960 Broadway, New York, NY 10032 USA; 2 Department of Medicine, Columbia University Irving Medical Center, New York, USA; 3 Division of Infectious Diseases, Columbia University Irving Medical Center, New York, USA; 4 Division of Pediatric Infectious Diseases, Columbia University Irving Medical Center, New York, USA; 5 Division of Pulmonary, Allergy, and Critical Care Medicine, Columbia University Irving Medical Center, New York, USA; 6 Division of Digestive and Liver Diseases, Columbia University Irving Medical Center, 630 West 168th Street, P&S 3–401, New York, NY 10032 USA

**Keywords:** Antimicrobial resistance, Antibiotics, Sepsis, Colonization, Healthcare-associated infection

## Abstract

**Background:**

The need for early antibiotics in the intensive care unit (ICU) is often balanced against the goal of antibiotic stewardship. Long-course antibiotics increase the burden of antimicrobial resistance within colonizing gut bacteria, but the dynamics of this process are not fully understood. We sought to determine how short-course antibiotics affect the antimicrobial resistance phenotype and genotype of colonizing gut bacteria in the ICU by performing a prospective cohort study with assessments of resistance at ICU admission and exactly 72 h later.

**Methods:**

Deep rectal swabs were performed on 48 adults at the time of ICU admission and exactly 72 h later, including patients who did and did not receive antibiotics. To determine resistance phenotype, rectal swabs were cultured for methicillin-resistant *Staphylococcus aureus* (MRSA) and vancomycin-resistant *Enterococcus* (VRE). In addition, Gram-negative bacterial isolates were cultured against relevant antibiotics. To determine resistance genotype, quantitative PCR (qPCR) was performed from rectal swabs for 87 established resistance genes. Within-individual changes in antimicrobial resistance were calculated based on culture and qPCR results and correlated with exposure to relevant antibiotics (e.g., did β-lactam antibiotic exposure associate with a detectable change in β-lactam resistance over this 72-h period?).

**Results:**

Of 48 ICU patients, 41 (85%) received antibiotics. Overall, there was no increase in the antimicrobial resistance profile of colonizing gut bacteria during the 72-h study period. There was also no increase in antimicrobial resistance after stratification by receipt of antibiotics (i.e., no detectable increase in β-lactam, vancomycin, or macrolide resistance regardless of whether patients received those same antibiotics). This was true for both culture and PCR. Antimicrobial resistance pattern at ICU admission strongly predicted resistance pattern after 72 h.

**Conclusions:**

Short-course ICU antibiotics made little detectable difference in the antimicrobial resistance pattern of colonizing gut bacteria over 72 h in the ICU. This provides an improved understanding of the dynamics of antimicrobial resistance in the ICU and some reassurance that short-course antibiotics may not adversely impact the stewardship goal of reducing antimicrobial resistance.

## Introduction

Empiric antibiotics are the main pillar of sepsis treatment in the intensive care unit (ICU). There is a 10–42% absolute increase in sepsis mortality when initial empiric antibiotics fail to appropriately treat infecting organisms [1–3]. Clinical guidelines recommend that broad-spectrum, empiric antibiotics be initiated as part of a treatment bundle within 1 h of presentation with sepsis [4], and studies suggest that outcomes may improve when antibiotics are given as quickly as possible [5].

Balanced against the imperative for early, broad-spectrum antibiotics is the mandate for antibiotic stewardship. Using fewer or more narrow-spectrum antibiotics avoids drug-drug interactions, avoids antibiotic side effects, and furthers the stewardship goal of minimizing the emergence of antimicrobial resistance [6].

Long courses of antibiotics in the ICU are associated with increased gastrointestinal antimicrobial resistance [7]. The impact of short-course antibiotics on antimicrobial resistance in the ICU is less certain. Large structural microbiome changes can be seen within 72 h after oral antibiotic intake in healthy, antibiotic-naïve volunteers [8]. Whether antimicrobial resistance can emerge so quickly following intravenous antibiotics in the ICU is not known.

This study gathered rectal swabs from ICU patients immediately at the time of ICU admission and 72 h later, and compared changes in gastrointestinal antimicrobial resistance in those who did or did not receive antibiotics among different classes. The goal of the study was to determine if short-term antibiotics adversely impact gastrointestinal antimicrobial resistance in the ICU.

## Methods

### Population

A random subset of 48 patients was selected from a previously described prospective cohort parent study [9, 10]. Adults ≥ 18 years old were eligible for the parent study if they were emergently admitted to the ICU from 2017 to 2019 at our institution, and if rectal swabs could be obtained within 4 h of ICU admission. The parent study gathered rectal swabs on patients at ICU admission and 72 h later (± 4 h). This 72-h timeframe represents a common minimum period after which antibiotic discontinuation might be considered [4]. For this study, 48 patients were selected using a random-picking algorithm from 179 patients within the parent study who had available sequenced rectal swabs from both ICU admission and 72 h later. All patients in the study had single rooms (no roommates) and gown and glove contact precautions were used for the duration of the study for patients with known MRSA, VRE, *C*. *difficile*, or extended-spectrum β-lactamase (ESBL) Gram-negative colonization. Informed consent was obtained from all subjects or from appropriate surrogates when subjects lacked capacity. The study was approved by the institutional review board of Columbia University.

### Study assessments

At each study assessment (ICU admission and 72 h later), samples were taken and clinical information was gathered. Two duplicate deep rectal flocked nylon swabs [11] were collected with the patient in the lateral decubitus position, with fecal staining of swabs used to verify adequate sampling. Demographic information, laboratory data, and data related to interventions performed in the ICU between study visits were extracted from the electronic medical record. For laboratory data, test results were used from the first venous blood draw in the ICU (corresponding to the first study assessment) and from a venous blood draw either at or immediately preceding the 72 h mark (corresponding to the second study assessment). ICU interventions were recorded including antibiotics, proton pump inhibitors, mechanical ventilation, hemodialysis, and enteral feeding. Clinical and laboratory data were used to estimate acute severity of illness according to the Sequential Organ Failure Assessment (SOFA) score as recommended by Sepsis-3 [12].

### Receipt of antibiotics

The study enrolled patients who did and did not receive antibiotics during the initial 72 h in the ICU. Use of antibiotics, and which antibiotics were used, was determined by treating ICU teams based on clinical need, without reference to the study. Receipt of antibiotics was classified categorically, without respect to the number of doses or route of administration, based on whether antibiotics were received in the ICU between the initial admission rectal swab and the 72-h rectal swab. For this study, antibiotics were considered broad-spectrum if they fell within the following class categories: β-lactam/β-lactamase inhibitor combination antibiotics, carbapenems, cephalosporins, fluoroquinolones, and lincosamides. This definition covers 5 of the 7 most commonly prescribed classes of antibiotics in US hospitals [13]. The term β-lactams has been used to describe ampicillin, β-lactam/β-lactamase combination antibiotics, carbapenems, and cephalosporins.

### Resistance phenotype

Resistance phenotype was determined using selective and non-selective cultures. Rectal swabs were inoculated into soy broth with 20% glycerol media at the bedside. After gentle mixing, these swabs were plated on 3 media: (1) selective chromogenic media for VRE including *E*. *faecalis* or *E*. *faecium*, (2) selective chromogenic media for MRSA, and (3) MacConkey II agar for Gram-negative bacteria. All plates were incubated aerobically at 33–37 °C and assessed after > 24 h. VRE and MRSA were classified as present versus absent according to the manufacturer’s instructions. Resistance phenotype for Gram-negative isolates was determined using the VITEK 2 system and AST-N010/020 cards with confirmatory testing as needed. Routine Clinical and Laboratory Standards Institute (CLSI) cut-offs were used for non-susceptibility [14].

### Resistance genotype

Resistance genotype was determined using a quantitative PCR kit that assesses 87 common antibiotic resistance genes (complete list of genes in Supplemental Table 1, Qiagen Cat. No. 330261) [15]. To do this, DNA was extracted from the duplicate rectal swab and 250 ng of template meta-genomic DNA was added to each reaction on a 96-well RT-PCR plate and run according to the manufacturer’s protocol. Controls were used to detect the presence of bacterial DNA, PCR inhibitors, and background. Adequate reactions were determined by cycle thresholds (*C*_T_) values of < 29 for the pan-bacterial reference genes *16S rRNA*, *gyrA*, *recA*, and *rpoB* and positive PCR control *C*_T_ values of < 24 [16]. Genotype was classified both as a continuous variable based on *C*_T_ value and also, per assay recommendations, as a categorical variable with *C*_T_ values of ≤ 34 considered positive for the presence of a given gene.

### Statistical approach

Summary data was compared using chi-squared tests or Fisher’s test when cell counts were < 5. For continuous summary data, *t* tests or rank-sum tests were used when the data was not normal in distribution. Chi-squared or Fisher’s tests were used to compare resistance-related outcomes that were classified categorically; patients who already showed resistance at ICU admission were excluded from such testing, because they did not have the possibility of developing new colonization. The final sample size of 48 patients gave 80% power to detect a difference in paired means representing antimicrobial resistance genotype of 0.41 standard deviations, with resistance genotype classified as a continuous variable based on *C*_T_ value. All testing was done two-sided at an alpha 0.05 level of significance using R.

## Results

### Population

A total of 48 critically ill patients were included in the study and swabbed at the time of ICU admission and 72 h later (Table 1). This 72-h window was selected because antibiotic discontinuation in the ICU is often first considered after 72 h of antibiotic treatment. Median sequential organ failure (SOFA) score was 16 (IQR, 10–18) at ICU admission and 17 (IQR, 15–19) after 72 h (Supplemental Table 2). Raw data for the study is given in Data Supplement 1 (phenotype) and Data Supplement 2 (genotype).

**Table 1:** Baseline characteristics of the patients in the study, treatments received in the ICU, and clinical outcomes within 30 days

Baseline characteristic	***N*** (%), ***N*** total = 48
Age (median years, IQR)	64 (52–74)
Female	21 (44%)
Admitted to ICU from hospital floor	12 (25%)
Baseline immunosuppression	18 (38%)
Primary reason for ICU admission, organized by organ system	
Cardiovascular/shock	14 (29%)
Respiratory failure	10 (21%)
Neurological	7 (15%)
Gastrointestinal	6 (13%)
Liver	5 (10%)
Malignancy	3 (6%)
Renal failure	3 (6%)
**Treatments received in the ICU, from the time of admission until 72 h later**	
Antibiotics	
Any antibiotics	41 (85%)
Broad-spectrum antibiotics	39 (81%)
Non-antibiotic interventions	
Enteral feeding	36 (75%)
Opioids	35 (73%)
Mechanical ventilation	26 (54%)
Proton pump inhibitors	22 (46%)
Hemodialysis	6 (13%)
Clinical outcomes within 30 days of ICU admission*	
Culture-proven infections	19 (40%)
MDR infections	14 (29%)
Death	11 (23%)

Immunosuppression was defined as a history of solid organ transplant or as a receipt of ablative chemotherapy, steroids at the equivalent of ≥ 5 mg/day prednisone, antimetabolites, anti-TNFα agents, calcineurin inhibitors, or mycophenolate. Broad-spectrum antibiotics were β-lactam/β-lactamase inhibitor combination antibiotics, cephalosporins, fluoroquinolones, lincosamides (clindamycin), and monobactams (e.g., meropenem)

*See reference [17] for operationalization of culture-proven infections; MDR infections were the subset of culture-proven infections caused by MRSA, VRE, and Gram-negative bacteria with non-susceptibility to 3rd-generation cephalosporins

### Receipt of antibiotics

**Fig. 1:**
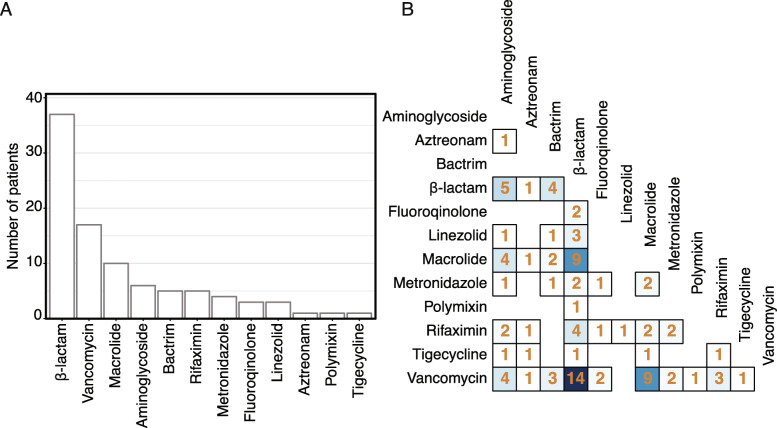
Receipt of antibiotics during the study. **a** Distribution of the number of patients receiving each antibiotic during the 72 h of the study. **b** Heatmap of pairwise antibiotic combinations. The numbers on the heatmap denote the number of patients receiving each drug pair during the 72 h of the study

Patients were eligible for inclusion in the study if they did or did not receive antibiotics. In sum, 41/48 (85%) of patients in the study received antibiotics and 37/48 (77%) received broad-spectrum antibiotics, most often a 3rd-generation cephalosporin or an extended-spectrum penicillin with a β-lactamase inhibitor. Figure 1a shows the antibiotics received by class, and Fig. 1b shows pairwise combinations of antibiotics. Almost all antibiotics were intravenous. Of 91 antibiotics dosed to the 48 patients, 93% were given intravenously (1 patient received oral azithromycin, 1 received oral vancomycin, and 4 received oral rifaximin).

### Overall changes in antimicrobial resistance phenotype and genotype

**Fig. 2:**
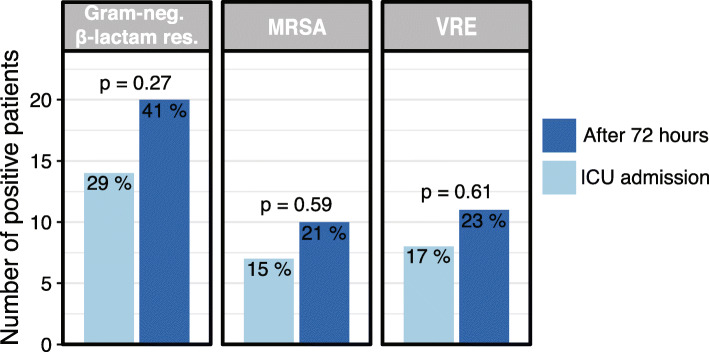
Incidence of antimicrobial resistance phenotype at ICU admission and 72 h later, based on culture for β-lactam resistance in Gram-negative bacteria, MRSA, and VRE. There was no significant increase in resistance after 72 h although there were trends in that direction. Chi-squared or Fisher’s *p* values are shown

First, antimicrobial resistance phenotype was examined by culturing rectal swabs and performing susceptibility testing. No differences were evident comparing summary data for resistance phenotype at ICU admission versus 72 h later for MRSA (RR 1.4, 95% CI 0.6–3.4; *p* = 0.59), VRE (RR 1.4, 95% CI 0.6–3.1; *p* = 0.61), or Gram-negative bacteria showing β-lactam resistance (RR 1.4, CI 0.8–2.4; *p* = 0.27 (Fig. 2)). Summing all antimicrobial resistance phenotype categories, there were no differences in rates of antimicrobial resistance for Gram-negative bacteria comparing ICU admission (non-susceptibility for 88/960 antibiotics tested, 9.1%) versus 72 h later (non-susceptibility for 107/960 antibiotics tested, 11.1%) (chi-squared *p* = 0.17).

Next, antimicrobial resistance genotype was examined by performing qPCR across 87 common antibiotic resistance genes from the rectal swabs. No differences were evident comparing summary genotype data from ICU admission versus 72 h later for genes conferring resistance to β-lactams (chi-squared *p* = 0.27), vancomycin (*p* = 1.0), macrolides (*p* = 0.93), or fluoroquinolones (*p* = 0.74) (Supplemental Fig. 1). Summing all resistance genes, there were no differences in rates of antimicrobial resistance comparing ICU admission (positive qPCR for 392 of 4176 genes, 9%) versus 72 h later (positive qPCR for 421 of 4176 genes, 10%) (chi-squared *p* = 0.30).

### Effect of antibiotics on antimicrobial resistance phenotype

**Fig. 3:**
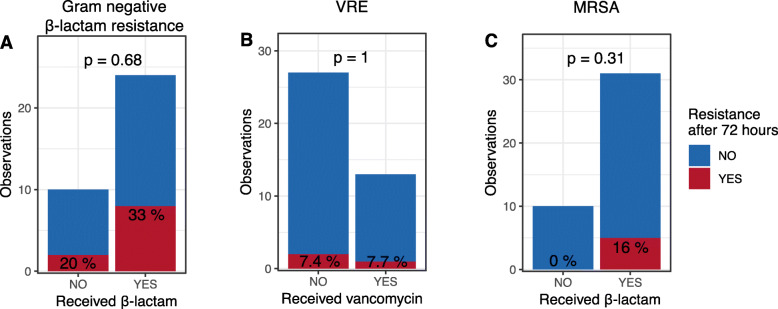
Change in antimicrobial resistance phenotype stratified by receipt of antibiotics. The vertical axis for each panel shows the number of patients who did (red) or did not (blue) test positive for resistance within an antibiotic class category. This data is then stratified on the horizontal axis by whether antibiotics within that same category were received (e.g., β-lactam resistance and receipt of β-lactam antibiotics). The panels are for **a** Gram-negative bacteria with β-lactam resistance, **b** vancomycin-resistant *Enterococcus* (VRE), and **c** methicillin-resistant *Staphylococcus aureus* (MRSA). In all panels, data is shown based on testing done after 72 h in the ICU for individuals that tested negative at admission. *p* values are for Fisher’s test, comparing resistance after 72 h based on receipt of antibiotics within the relevant category

For patients that did not carry individual resistant bacteria at admission, the carriage rate after 72 h for those that received relevant antibiotics was compared to the carriage rate for those that did not receive relevant antibiotics (e.g., comparing β-lactam non-susceptibility in culture based on receipt of β-lactam antibiotics). Antibiotics had no significant association with resistance phenotype (Fig. 3). Emergence of Gram-negative bacteria showing resistance to at least one β-lactam antibiotic after 72 h was seen in 8/24 (33%) of patients who received β-lactam antibiotics and in 2/10 (20%) of patients who did not (RR 1.7, 95% CI 0.43–6.51; Fisher’s *p* = 0.68). VRE was present in 1/13 (8%) patients who received vancomycin and in 2/27 (7%) of patients who did not (RR 1.0, 95% CI 0.10–10.4; *p* = 1.0). MRSA was present in 5/31 (16%) patients who received β-lactams and in 0/10 (0%) of patients who did not (RR unable; *p* = 0.31). No other clinical interventions (enteral feeding, opioids, mechanical ventilation, and proton pump inhibitors) associated with detectable differences in resistance phenotype.

### Effect of antibiotics on antimicrobial resistance genotype

**Fig. 4:**
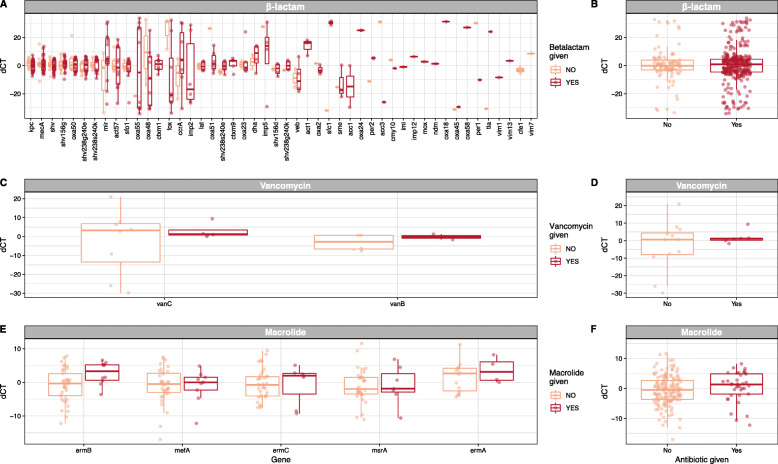
Within-individual changes in antimicrobial resistance genotype are shown stratified by receipt of antibiotics. Rows are organized by antibiotic classes and columns show data for individual genes (left panels) and then summary data (right panels): **a** 48 β-lactam resistance genes and **b** combined data across all 48 genes; **c** 2 vancomycin resistance genes (*vanC* and *vanB*) and **d** combined data across the 2 genes; and **e** 5 macrolide resistance genes and **f** combined data across the 5 genes. Data has been calculated based on the difference in PCR *C*_T_ values from admission to 72 h (i.e., admission *C*_T_ value minus 72-h *C*_T_ value for each gene). The within-individual dC_T_ values are displayed as raw data with an overlay of box-and-whisker plots. None of the differences was statistically significant, comparing dC_T_ values for those who received antibiotics within the relevant category to those who did not (e.g., comparing dC_T_ values for β-lactam antibiotics for patients who received β-lactam antibiotics versus patients who did not receive β-lactam antibiotics)

Change in antimicrobial resistance gene abundance was tested after stratifying by receipt of antibiotic class (Fig. 4). This was first done with a change in genotype classified as a continuous variable based on within-individual change in *C*_T_ values (ICU admission *C*_T_ minus 72-h *C*_T_). There was no association between the changes within relevant resistance genes after 72 h and receipt of any of the 3 most common antibiotic categories (β-lactams, vancomycin, or macrolides). There was also no overall difference in the within-individual change in *C*_T_ values comparing combined antibiotic gene categories (*t* test *p* = 0.49 for β-lactams, *p* = 0.28 for vancomycin, and *p* = 0.19 for macrolides) (Fig. 4). This analysis was then repeated classifying within-individual change in genotype categorically (i.e., present versus absent). Again, there was no association between receipt of antibiotics and changes within relevant antibiotic resistance genotypes. Last, other ICU interventions were examined. Opioids were associated with modestly reduced within-individual risk of an increase in combined genotype (RR 0.79, 95% CI 0.69–0.91; *p* < 0.01). No other clinical interventions associated with detectable differences in resistance genotype.

### ICU admission antimicrobial resistance pattern as a predictor of resistance phenotype and genotype after 72 h

**Fig. 5:**
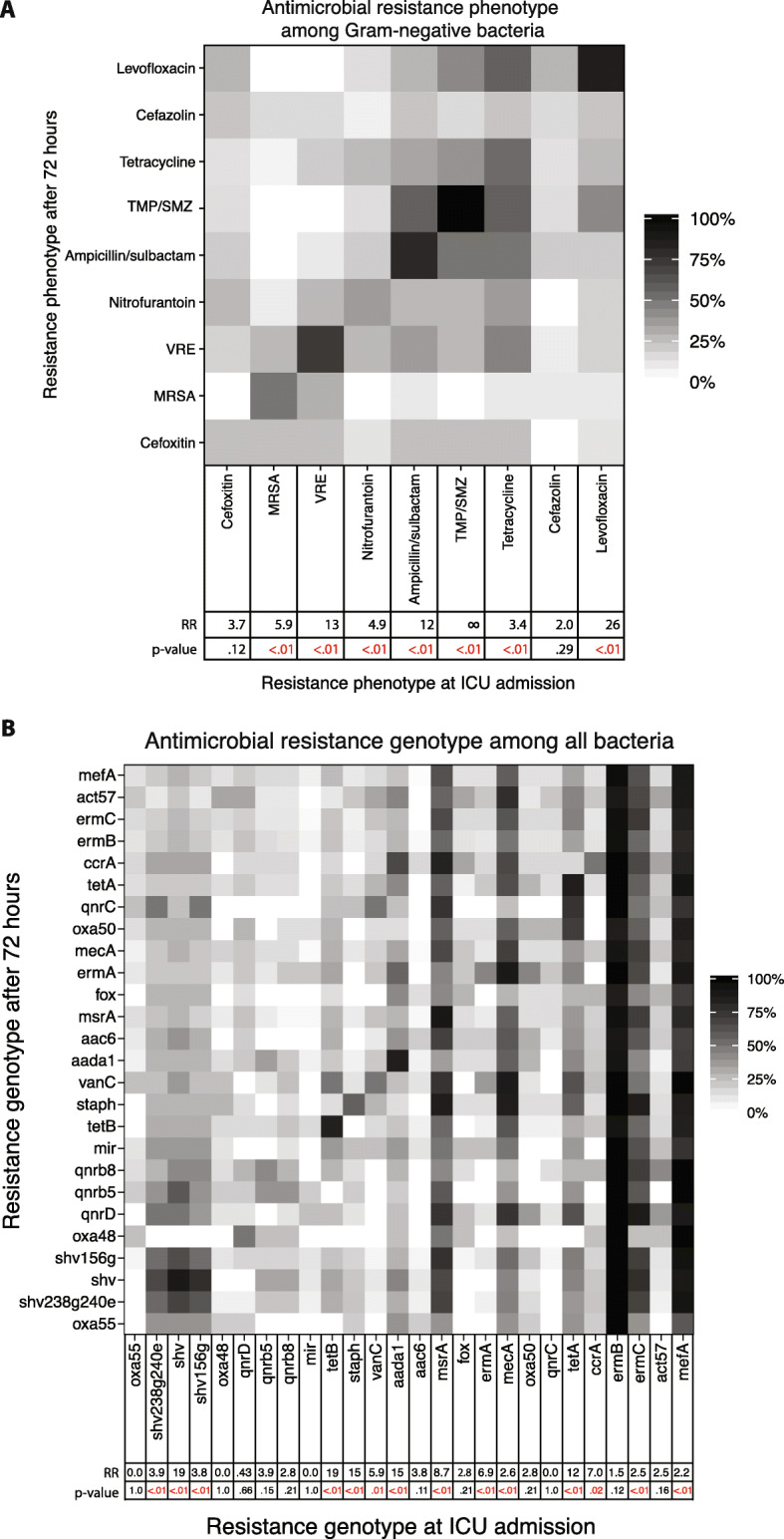
Relationship between individuals’ resistance pattern at ICU admission compared to 72 h later, for antimicrobial resistance phenotype and genotype. **a** Heatmap depicting the percentage of patients with resistant isolates at 72 h that also had resistant isolates at admission. **b** Heatmap depicting the percentage of patients with resistance genes at 72 h that also had resistance genes at admission. For both heatmaps, horizontal axes are resistance at admission and vertical axes are resistance after 72 h. Bottom rows show (1) relative risk (RR) of testing positive for a resistance category at 72 h for those that were resistant to the same category at admission versus those who tested negative on admission and (2) *p* values for the same comparison. In panel **b**, the cases of RR = 0 reflect that no sample-pairs were positive on both admission and 72 h later

Last, the ICU admission antimicrobial resistance pattern was examined as a predictor of resistance after 72 h for both phenotype and genotype. For 7 of the 9 antimicrobial resistance categories tested, presence of the resistant phenotype (i.e., non-susceptibility in culture) at the time of ICU admission was significantly associated with non-susceptibility 72 h later (Fig. 5a). For 14 of the 26 genes tested, presence of the resistance genotype (i.e., positive qPCR) at the time of ICU admission was significantly associated with positive qPCR 72 h later (Fig. 5b).

## Discussion

In this study of 48 critically ill patients, there was no association between receipt of short-course antibiotics and change in antimicrobial resistance phenotype or genotype during the 72 h following ICU admission. Initial patient-level ICU antimicrobial resistance pattern was the best predictor of antimicrobial resistance after 72 h, and significant interim changes were rare. Opioids, which have traditionally been associated with poor ICU outcomes, were associated with modestly reduced overall resistance in antimicrobial genotype.

This study addressed the question of short-term resistance dynamics within the gut microbiome of ICU patients. Clinically, the imperative for early broad-spectrum antibiotics in the ICU is balanced against the desire for antibiotic stewardship. Decisions regarding use of antibiotics for sepsis are usually made before diagnostic microbiology results are available [18, 19]. Can intensivists reasonably give and continue empiric broad-spectrum antibiotics for 72 h without excessive concern that they are promoting antimicrobial resistance? Our results are reassuring but must be interpreted with caution. Prior studies clearly establish that antimicrobial resistance arises within colonizing gut bacteria during long-term antibiotic treatment in the ICU. The question is not *if* resistance develops but *when*. The 72-h treatment window in this study parallels the 3-day interval after which antibiotic discontinuation is sometimes considered in the ICU [4, 20]. The results suggest that 72 h is not long enough for the development of new gastrointestinal antimicrobial resistance within a given individual. Longer antibiotic treatment window periods would almost certainly have led to different study results.

These findings regarding the dynamics of resistance contrast sharply with in vitro studies. When bacterial isolates are exposed to selective pressure in culture, mutations that confer antimicrobial resistance are rapidly selected [21, 22]. Compared to in vitro systems, the gut microbiome of ICU patients has many competing selection pressures. This dense network of interactions may delay selection for antimicrobial resistance genes [23]. In ICU patients, collapse of the pre-existing gut microbiome and emergence of a pathobiome enriched in resistance may require 11–14 days of antibiotic treatment [24, 25]. Other studies suggest that resistance does emerge, but does so slowly. In allogeneic stem cell transplant patients, emergence of new resistance within multiple VRE clones was seen after 7 days of selective antibiotics, with most new resistance observed after 3 weeks or more [26]. In an infant treated with multiple antibiotics, 2 months were required before antimicrobial resistance emerged within specific bacterial lineages [27, 28]. In a similar study, novel plasmid-mediated ampicillin resistance was acquired after 16–32 days in the absence of antibiotic treatment [29]. The implication is that resistance within complex human systems such as the gut arises over weeks instead of days [7].

Multiple factors probably contributed to the relatively modest changes observed in antimicrobial resistance. The antibiotics received were 93% intravenous. Intravenous antibiotics do penetrate into the gut [30], but luminal concentrations and pharmacodynamics may matter [31]. Another possibility is that patients were already too enriched in antimicrobial resistance at the time of ICU admission because of past antibiotic exposures to detect a meaningful change in resistance over 72 h. Prior studies support such a conclusion. Willmann et al. found surprisingly little gains in fluoroquinolone resistance during prophylaxis of neutropenic patients, perhaps because of past exposures [32].

This study has limitations. It did not seek to correlate antimicrobial resistance with specific bacterial lineages and cannot state whether “new” antimicrobial resistance was acquired from the environment, from horizontal gene transfer, or vertically within bacterial lineages. Such correlations are technically challenging [33]. Rather, a standard clinical culture-based approach was used to identify resistance within the primarily Gram-negative bacteria that cause most serious ICU infections [34]. Then quantitative PCR was added to determine resistance genotype. Alternative methodologies could have been used for genotyping [35], but qPCR was selected for ease of performance and high sensitivity [36]. The ICUs involved were high-acuity regional referral centers, and results may not generalize perfectly to other ICUs. Last, the study was relatively small. While there were within-individual increases in antibiotic resistance based on culture (see Fig. 2), the study was not powered to detect weak relationships between antibiotics and antimicrobial resistance, especially for certain antibiotic class categories where few patients were unexposed. Given the large historical benefits attributed to antibiotics, a modest effect on antimicrobial resistance is unlikely to significantly alter the clinical risk-benefit calculation.

## Conclusions

In sum, no clear relationship could be detected between receipt of antibiotics and antimicrobial resistance within colonizing gut bacteria during the initial 72 h in the ICU. This was the case for resistance phenotype based on culture and resistance genotype based on qPCR. Antimicrobial resistance was relatively stable between ICU admission and the 72 h mark. This result may provide some reassurance that short-course antibiotics given at ICU admission do not necessarily have an adverse effect on individuals’ antimicrobial resistance.

## Supplementary information

**Additional file 1: Table S1.** Antibiotic resistance genes tested with qPCR, organized by category.

**Additional file 2: Table S2.** Sequential Organ Failure Assessment (SOFA) characteristics and scores, calculated at the time of ICU admission and 72 hours later.

**Additional file 3: Figure S1.** Incidence of antimicrobial resistance genotype at ICU admission and 72 hours later based on qPCR. The resistance genes are grouped by the antibiotic class they confer resistance to. Samples were considered positive for a given gene if the C_T_ value was ≤ 34, There was no significant increase in resistance genotype after 72 hours. Chi-squared or Fisher’s *p*-values are shown.

**Additional file 4.**

**Additional file 5.**

## Data Availability

Complete raw data for the study has been made available as an online supplement at 10.1186/s13054-020-03061-8.

## Abbreviations

ICU: Intensive care unit
IQR: Interquartile range
MDR: Multidrug resistant
MRSA: Methicillin-resistant *Staphylococcus aureus*
PCR: Polymerase chain reaction
VRE: Vancomycin-resistant *Enterococcus*
