# Supplementary material for: The effect of short-course antibiotics on the resistance profile of colonizing gut bacteria in the ICU: a prospective cohort study
Source: Crit Care. 2020 Jul 9;24:404. doi: 10.1186/s13054-020-03061-8 (PMC7350675; doi:10.1186/s13054-020-03061-8)
Supplement: Supplementary file 1 — Additional file 1: Table S1. Antibiotic resistance genes tested with qPCR, organized by category. [file 13054_2020_3061_MOESM1_ESM.docx]

**Supplemental Table 1.** Antibiotic resistance genes tested with qPCR, organized by category.

| **Category** | **Genes** |
| --- | --- |
| Aminoglycoside | *aacC1, aacC2, aacC4, aadA1, apha6* |
| Class A beta-lactamase | *BES-1, BIC-1, CTX-M-1, CTX-M-8, CTX-M-9, GES, IMI & NMC-A, KPC, Per-1, Per-2, SFC-1, SFO-1, SHV (156D) (156G) (238G240E) (238G240K) (238S240E) (238S240K), SME, TLA-1, VEB* |
| Class B beta-lactamase | *ccrA, IMP-1, IMP-12, IMP-2, IMP-5, NDM, VIM-1, VIM-13, VIM-7* |
| Class C beta-lactamase | *ACC-1, ACC-3, ACT 5/7, ACT-1, CFE-1, CMY-10, DHA, FOX, LAT, MIR, MOX* |
| Class D beta-lactamase | *OXA-10, OXA-18, OXA-2, OXA-23, OXA-24, OXA-45, OXA-48, OXA-50, OXA-51, OXA-54, OXA-55, OXA-58, OXA-60* |
| Erythromycin | *ereB* |
| Fluoroquinolone | *AAC(6)-Ib-cr, QepA, QnrA, QnrB-1, QnrB-31, QnrB-4, QnrB-5, QnrB-8, QnrC, QnrD, QnrS* |
| Macrolide-Lincosamide | *ermA, ermB, ermC, mefA, msrA* |
| MDR efflux pump | *oprj, oprm* |
| Tetracycline | *tetA, tetB* |
| Beta-lactam | *mecA* |
| Vancomycin | *vanB, vanC* |
| PVL chain F | *lukF* |
| IgG binding protein A | *spa* |
