# Supplementary material for: The effect of short-course antibiotics on the resistance profile of colonizing gut bacteria in the ICU: a prospective cohort study
Source: Crit Care. 2020 Jul 9;24:404. doi: 10.1186/s13054-020-03061-8 (PMC7350675; doi:10.1186/s13054-020-03061-8)
Supplement: Supplementary file 2 — Additional file 2: Table S2. Sequential Organ Failure Assessment (SOFA) characteristics and scores, calculated at the time of ICU admission and 72 hours later. [file 13054_2020_3061_MOESM2_ESM.docx]

**Supplemental Table 2**. Sequential Organ Failure Assessment (SOFA) characteristics and scores, calculated at the time of ICU admission and 72 hours later.

| **SOFA characteristics** | **ICU admission**  **N (%)** | **72 hours**  **N (%)** |
| --- | --- | --- |
| Respiration: Ventilator support at the time of assessment | 26 (54%) | 14 (29%) |
| Respiration: PaO_2_/FiO_2_ < 400 or < 200 with ventilator support | 20 (42%) | 9 (19%) |
| Coagulation: platelet count < 150 x 10^3^/mm^3^ | 28 (58%) | 25 (52%) |
| Liver: serum bilirubin > 1.2 mg/dL | 11 (23%) | 14 (29%) |
| Shock: mean arterial pressure < 70 mm Hg or use of vasopressors | 22 (46%) | 19 (40%) |
| Neurologic: Glasgow coma scale < 15 points* | 26 (54%) | 19 (40%) |
| Renal: Serum creatinine > 1.2 mg/dL or urine output < 500 mL/day** | 24 (50%) | 28 (58%) |
| **Total SOFA points**  **(median, IQR)** | **16 (10-18)** | **17 (15-19)** |

SOFA criteria were calculated based on the worst values during the preceding 24 hours. *Glasgow coma scale (GCS) was the assumed GCS absent sedating medications. **SOFA urine output criteria was not applied for patients with baseline end-stage renal disease (n=2).
