# Supplementary figures and images for: The effect of short-course antibiotics on the resistance profile of colonizing gut bacteria in the ICU: a prospective cohort study
Source: Crit Care. 2020 Jul 9;24:404. doi: 10.1186/s13054-020-03061-8 (PMC7350675; doi:10.1186/s13054-020-03061-8)

Number of positive genes

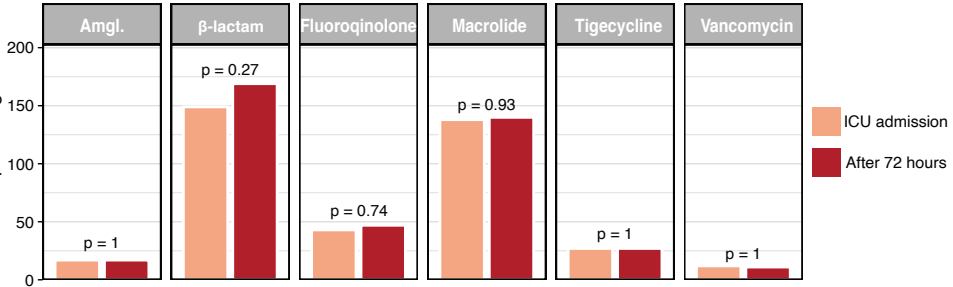

Supplement: Supplementary file 3 — Additional file 3: Figure S1. Incidence of antimicrobial resistance genotype at ICU admission and 72 hours later based on qPCR. The resistance genes are grouped by the antibiotic class they confer resistance to. Samples were considered positive for a given gene if the CT value was ≤ 34, There was no significant increase in resistance genotype after 72 hours. Chi-squared or Fisher’s p-values are shown. [file 13054_2020_3061_MOESM3_ESM.pdf]
